# Supplementary material for: Risk-Stratified Breast Cancer Screening Incorporating a Polygenic Risk Score: A Survey of UK General Practitioners’ Knowledge and Attitudes
Source: Genes (Basel). 2023 Mar 16;14(3):732. doi: 10.3390/genes14030732 (PMC10048009; doi:10.3390/genes14030732)
Supplement: Supplementary file 1 [file genes-14-00732-s001.zip › genes-2128189-supplementary.pdf]

Supplementary Data

Supplementary Material Text S1. A copy of the online survey

Imperial College London

Participant Information

An anonymous survey of UK GPs' knowledge and attitudes towards the use of polygenic risk scores in breast cancer screening

Researcher: Dr Aya Ayoub, MSc Genomic Medicine student, Imperial College London aya.ayoub20@imperial.ac.uk

As a qualified GP or GP trainee practicing in the UK, you are invited to complete this questionnaire which forms part of my MSc Genomic Medicine at Imperial College London. The aim of this questionnaire is to assess UK GPs' knowledge and attitudes towards polygenic risk scores (PRS) *for risk stratification in breast cancer screening.*

What is the purpose of this study?

PRS quantifies the cumulative effects of multiple common genetic variants - single nucleotide polymorphisms, 'SNPs' - which individually have a low effect on disease susceptibility but, when combined, have been shown to substantially increase an individual's risk of developing breast cancer.

Use of PRS to predict risk of future breast cancer is currently being evaluated within the NHS through the 'CanRisk' online risk-assessment tool. 'CanRisk' offers a personalised breast cancer risk assessment *by combining a PRS for 313 SNPs (validated in women of white* European ancestry only) with testing for rarer high-impact pathogenic variants in cancer predisposing genes (e.g. *BRCA1*) and non-genetic factors including family history, mammographic density, lifestyle and hormonal risk factors.

Why have I been invited?

You have been invited because you are a UK trainee or qualified GP. In the future PRS could be offered beyond specialist genetics clinics, and potentially incorporated into the UK national breast screening programme which currently offers 3-yearly mammographic screening to all women aged 50 – 71 years regardless of individual risk. Using PRS to risk-stratify individuals for future breast cancer could offer a more targeted approach to screening with the potential to reduce mortality in high-risk groups, reduce overdiagnosis and improve the cost-effectiveness of screening.

One of the many challenges to future PRS implementation is workforce attitudes and training. Patients being offered testing will particularly look to their GP for information and advice.

Do I have to take part?

This questionnaire should take no more than 10 minutes to complete. Participation is voluntary and responses are anonymous. Prior to submission, you are free to withdraw from the questionnaire at any time, without giving any reason.

What are the possible disadvantages and risks of taking part?

This is an online survey, therefore there are no disadvantages or risks associated with participation in this study.

What are the possible benefits of taking part

Results of the survey will be used to evaluate GPs' level of confidence with genomic concepts underpinning PRS which is necessary to understand and communicate the potential benefits and harms of testing to patients.

What will happen to the results of the research study?

Results of the survey will be evaluated as part of the researcher's MSc project, and may be published in a scientific journal following this. The survey is anonymised so participants will not be identified in any report/publication.

Who is organising and funding the research?

Imperial College London is the study sponsor. This study is unfunded.

Who has reviewed the study?

This study was given approval by the Head of Department and Research Governance Integrity Team (RGIT) at Imperial College London.

Contact for Further Information

Please contact the researcher for any queries or complaints: Dr Aya Ayoub, aya.ayoub20@imperial.ac.uk

Thank you for taking part in this study!

A copy of the written information can be printed online for you to keep.

Please tick the box if you agree to take part in this anonymous study

- I consent to take part in this study

1. Where are you in your GP career?

- GP Trainee
- Less than 5 years post-CCT
- 5-9 years post-CCT
- 10-14 years post-CCT
- 15-19 years post-CCT
- 20-24 years post-CCT
- 25+ years post-CCT

2. Which term most closely describes your role?

- Trainee GP
- Salaried GP
- GP Partner
- Locum GP
- Other (please specify)

3. Where do you work in the UK?

- East of England
- East Midlands
- London
- North East
- North West
- Northern Ireland
- Scotland
- South East

- South West
- Wales
- West Midlands
- Yorkshire and The Humber

4. What is your gender?
    - Male
    - Female
    - Non-binary / Other
    - Prefer not to say

5. Which ethnicity best describes you?

- **Asian or Asian British**
    - Indian
    - Pakistani
    - Bangladeshi
    - Chinese
    - Any other Asian background
- **Black, Black British, Caribbean or African**
    - Caribbean
    - African
    - Any other Black, Black British, or Caribbean background
- **Mixed or multiple ethnic groups**
    - White and Black Caribbean
    - White and Black African
    - White and Asian
    - Any other Mixed or multiple ethnic background

- **White**
    - English, Welsh, Scottish, Northern Irish or British
    - Irish
    - Gypsy or Irish Traveller
    - Roma
    - Any other White background
- **Other ethnic group**
    - Arab
    - Any other ethnic group
- **Prefer not to say**

6. How strongly do you agree or disagree that the current UK National Breast Screening programme, which offers 3-yearly mammograms to all women aged 50-71 years old, is an effective method for early detection of breast cancer?
    - Strongly agree
    - Somewhat agree
    - Neither agree nor disagree
    - Somewhat disagree
    - Strongly disagree

7. Using the sliding scale below, please rate your level of familiarity with PRS.

Not familiar		Slightly familiar		Moderately familiar		Very familiar	Extremely familiar
**0**	**1**	**2**	**3**	**4**	**5**	**6**	**7**

8. PRS provides a measure of the inheritance of many low-impact disease-associated SNPs that have been shown to correlate with - rather than cause - disease through large studies, 'genome wide association studies', that compare the genomes of patients with disease to those of healthy controls. On the other hand, high impact variants in - monogenic - cancer predisposing genes (e.g. *BRCA1*) are shown to cause disease by altering a gene's function, and have a clear mode of inheritance (e.g. autosomal dominant). Using the sliding scale below, please rate your level of confidence explaining the difference between a polygenic and monogenic condition to a patient.

Not confident [illegible] [illegible] [illegible] [illegible] [illegible]

0 1 2 3 4 5 6 7

9. Using the sliding scale below, please rate how confident you feel counselling a patient on the advantages and disadvantages of a personalised breast cancer risk assessment incorporating PRS?

Not confident Slightly confident Moderately confident Very confident Extremely confident

0 1 2 3 4 5 6 7

10. A personalised breast cancer risk assessment aims to offer a more targeted screening approach based on individual risk. Please indicate how strongly you agree or disagree with the following recommendations:

A personalised breast cancer risk assessment aims to offer a more targeted screening approach based on individual risk. Please indicate how strongly you agree or disagree with the following recommendations:

	**Strongly disagree**	**Somewhat disagree**	**Neither agree nor disagree**	**Somewhat agree**	**Strongly agree**
**For women who are at high risk of breast cancer, start screening at an earlier age**	For women who are at high risk of breast cancer , start screening at an earlier age Strongly disagree	For women who are at high risk of breast cancer , start screening at an earlier age Somewhat disagree	For women who are at high risk of breast cancer , start screening at an earlier age Neither agree nor disagree	For women who are at high risk of breast cancer , start screening at an earlier age Somewhat agree	For women who are at high risk of breast cancer , start screening at an earlier age Strongly agree
**For women who are at high risk of breast cancer, increase the frequency of screening**	For women who are at high risk of breast cancer , increase the frequency of screening Strongly disagree	For women who are at high risk of breast cancer , increase the frequency of screening Somewhat disagree	For women who are at high risk of breast cancer , increase the frequency of screening Neither agree nor disagree	For women who are at high risk of breast cancer , increase the frequency of screening Somewhat agree	For women who are at high risk of breast cancer , increase the frequency of screening Strongly agree

A personalised breast cancer risk assessment aims to offer a more targeted screening approach based on individual risk. Please indicate how strongly you agree or disagree with the following recommendations:

	**Strongly disagree**	**Somewhat disagree**	**Neither agree nor disagree**	**Somewhat agree**	**Strongly agree**
**For women who are at high risk of breast cancer, increase the frequency of screening and start at an earlier age**	For women who are at high risk of breast cancer , increase the frequency of screening and start at an earlier age Strongly disagree	For women who are at high risk of breast cancer , increase the frequency of screening and start at an earlier age Somewhat disagree	For women who are at high risk of breast cancer , increase the frequency of screening and start at an earlier age Neither agree nor disagree	For women who are at high risk of breast cancer , increase the frequency of screening and start at an earlier age Somewhat agree	For women who are at high risk of breast cancer , increase the frequency of screening and start at an earlier age Strongly agree
**For women who are at higher than average risk of breast cancer, increase the frequency of screening**	For women who are at higher than average risk of breast cancer , increase the frequency of screening Strongly disagree	For women who are at higher than average risk of breast cancer , increase the frequency of screening Somewhat disagree	For women who are at higher than average risk of breast cancer , increase the frequency of screening Neither agree nor disagree	For women who are at higher than average risk of breast cancer , increase the frequency of screening Somewhat agree	For women who are at higher than average risk of breast cancer , increase the frequency of screening Strongly agree
**For women who are at higher than average risk of breast cancer, increase the frequency of screening and start at an earlier age**	For women who are at higher than average risk of breast cance r, increase the frequency of screening and start at an earlier age Strongly disagree	For women who are at higher than average risk of breast cance r, increase the frequency of screening and start at an earlier age Somewhat disagree	For women who are at higher than average risk of breast cance r, increase the frequency of screening and start at an earlier age Neither agree nor disagree	For women who are at higher than average risk of breast cance r, increase the frequency of screening and start at an earlier age Somewhat agree	For women who are at higher than average risk of breast cance r, increase the frequency of screening and start at an earlier age Strongly agree
**For women who are at lower than average risk of breast cancer, start screening at a later age**	For women who are at lower than average risk of breast cancer , start screening at a later age Strongly disagree	For women who are at lower than average risk of breast cancer , start screening at a later age Somewhat disagree	For women who are at lower than average risk of breast cancer , start screening at a later age Neither agree nor disagree	For women who are at lower than average risk of breast cancer , start screening at a later age Somewhat agree	For women who are at lower than average risk of breast cancer , start screening at a later age Strongly agree
**For women who are at lower than average risk of breast cancer, decrease the frequency screening**	For women who are at lower than average risk of breast cancer , decrease the	For women who are at lower than average risk of breast cancer , decrease the	For women who are at lower than average risk of breast cancer , decrease the	For women who are at lower than average risk of breast cancer , decrease the	For women who are at lower than average risk of breast cancer , decrease the

A personalised breast cancer risk assessment aims to offer a more targeted screening approach based on individual risk. Please indicate how strongly you agree or disagree with the following recommendations:

	**Strongly disagree**	**Somewhat disagree**	**Neither agree nor disagree**	**Somewhat agree**	**Strongly agree**
	frequency screening Strongly disagree	frequency screening Somewhat disagree	frequency screening Neither agree nor disagree	frequency screening Somewhat agree	frequency screening Strongly agree
**For women who are at lower than average risk of breast cancer, decrease the frequency of screening and start at a later age**	For women who are at lower than average risk of breast cancer , decrease the frequency of screening and start at a later age Strongly disagree	For women who are at lower than average risk of breast cancer , decrease the frequency of screening and start at a later age Somewhat disagree	For women who are at lower than average risk of breast cancer , decrease the frequency of screening and start at a later age Neither agree nor disagree	For women who are at lower than average risk of breast cancer , decrease the frequency of screening and start at a later age Somewhat agree	For women who are at lower than average risk of breast cancer , decrease the frequency of screening and start at a later age Strongly agree
**For women who are at much lower than average risk of breast cancer, do not offer breast screening**	For women who are at much lower than average risk of breast cancer , do not offer breast screening Strongly disagree	For women who are at much lower than average risk of breast cancer , do not offer breast screening Somewhat disagree	For women who are at much lower than average risk of breast cancer , do not offer breast screening Neither agree nor disagree	For women who are at much lower than average risk of breast cancer , do not offer breast screening Somewhat agree	For women who are at much lower than average risk of breast cancer , do not offer breast screening Strongly agree

11. A personalised breast cancer risk assessment combines results for PRS, genetic and non-genetic risk factors to generate a 10-year absolute risk of having breast cancer e.g. 2.5% 10-year risk. Using the sliding scale below, please rate your level of confidence communicating a personalised breast cancer risk assessment result to a patient.

Not confident Slightly confident Moderately confident Very confident Extremely confident [illegible]

0 **1** **2** **3** **4** **5** **6** **7**

12. Using the sliding scale below, please rate the impact you feel a personalised breast cancer risk assessment would have on your patients.

Extremely negative		Somewhat negative		Neither agreed/disagree	Somewhat positive	Extremely positive	
**0**	**1**	**2**	**3**	**4**	**5**	**6**	**7**

13. Using the sliding scale below, please rate the impact you feel a personalised breast cancer risk assessment would have on your practice.

Extremely negative		Somewhat negative		Neither agreed/disagree	Somewhat positive	Extremely positive	
**0**	**1**	**2**	**3**	**4**	**5**	**6**	**7**

14. In your opinion, what aspects of the NHS should be enhanced to implement breast cancer screening based on a personalised risk assessment? (Check all that apply)

- Number of primary care physicians
- Number of nurse practitioners
- Number of genetic counsellors
- Number of geneticists
- Remuneration of healthcare professionals
- Training of healthcare professionals
- Time allocated to a patient-physician appointment
- Time allocated to a patient-nurse practitioner appointment
- Access to a primary care physician
- Access to a nurse or nurse practitioner
- Access to breast screening (e.g. mammogram, MRI)

- None, I believe the healthcare system is ready
- Other (please specify)

15. What type of information would you like to find in the resources you use in your clinical practice to better understand screening based on personalised risk assessment? (Check all that apply)

- General information on genetics
- Information on common genetic variants (SNPs)
- Information on the basics of personalised breast cancer risk assessment
- Information on the calculation of a polygenic risk score (PRS)
- Information on interpreting results of breast cancer risk assessment
- Information on the best practices of breast cancer risk level communication
- Information on breast cancer prevention
- Information on the main ethical, legal and social challenges of personalised breast risk assessment
- Other (please specify)

16. For learning more about breast cancer screening based on personalised risk assessment, please select the three resource formats you find most useful:

- In-person training such as workshops
- Online courses
- Webinar type conference
- Consultations with a geneticist or a genetic counsellor
- An application for your phone or tablet
- Printed material
- Website
- Other (please specify)

17. Finally, where did you find this questionnaire?

18. If you have any questions, comments or concerns please leave them here:

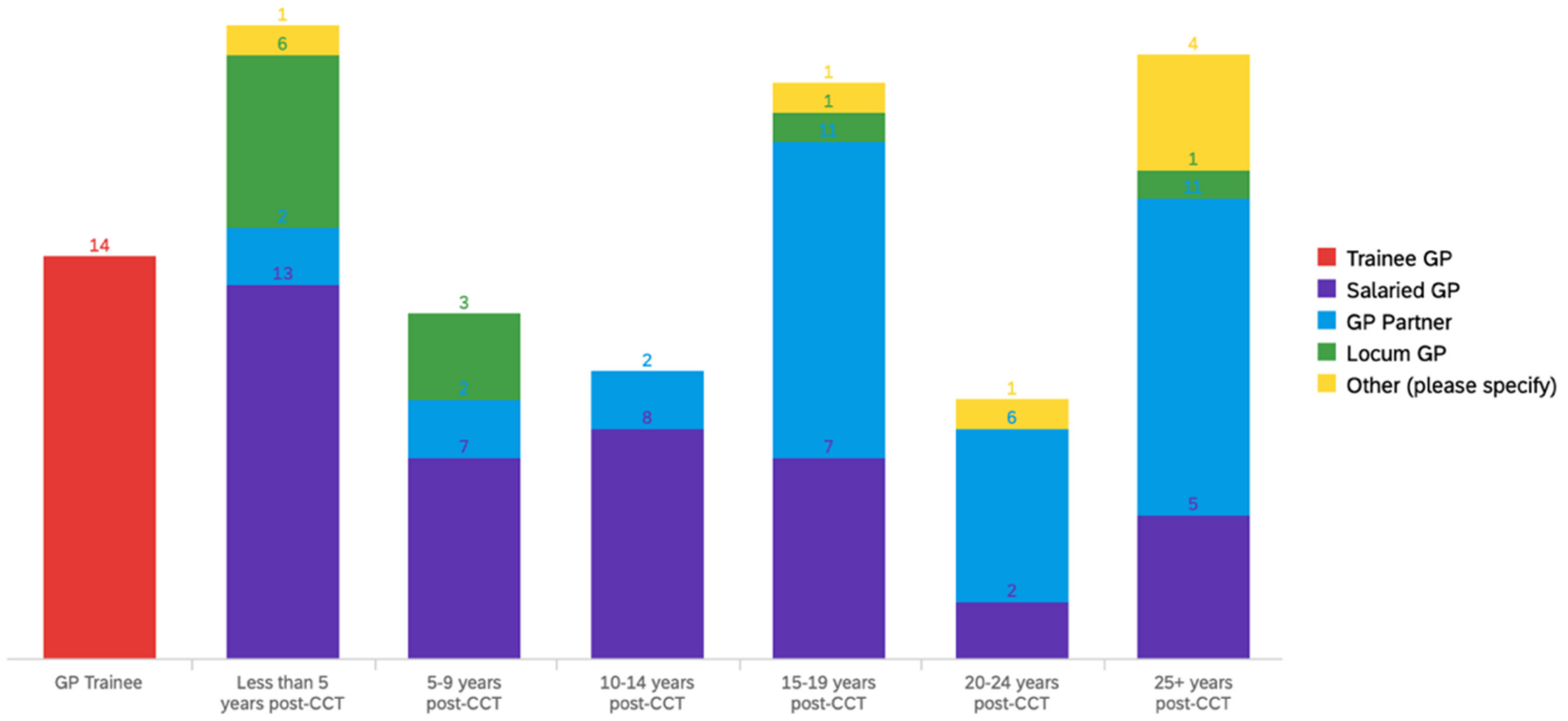

Figure S1. Stacked bar-chart showing the breakdown of respondents by years of practice and role
Bars are labelled with the frequency of responses per category.
'Other GPs' were: 4 academic GPs, 1 GP Educator, 1 recently retired GP, 1 GP retainer.
Overall, GP Partners had more years of practice than salaried and locum GPs.

Table S1. How strongly GPs agree/disagree that the current NHS Breast Screening Programme is an effective method for early detection of breast cancer

		Total Count (All)	Strongly or somewhat disagree		Neither agree nor disagree		Somewhat or strongly agree		p-value*
			N	%	N	%	N	%	
Years of GP experience	**Total**	108	17	16	12	11	79	73	
	GP Trainee	14	2	14	3	21	9	64	
	0 - 15 years post-CCT	44	7	16	7	16	30	68	
	15+ years post-CCT	50	8	16	2	4	40	80	0.09
GP Role	**Total**	109	17	16	12	11	80	73	
	Trainee GP	14	2	14	3	21	9	64	
	Qualified GP	95	15	16	9	9	71	75	0.20
Gender	**Total**	105	16	15	11	10	78	74	
	Male	43	3	7	8	19	32	74	
	Female	62	13	21	3	5	46	74	0.01
Location of practice	**Total**	109	17	16	12	11	80	73	
	London	38	3	8	6	16	29	76	
	Rest of UK	71	14	20	6	8	51	72	0.09
Ethnicity	**Total**	104	17	16	11	11	76	73	
	White	63	13	21	5	8	45	71	
	Non-white	41	4	10	6	15	31	76	0.12

*Bonferroni corrected p-value <0.003 is considered statistically significant

Tables S2. How strongly GPs agree/disagree with the following nine targeted screening approaches based on personalised breast cancer risk assessment:

1. **For women who are at high risk of breast cancer, start screening at an earlier age**

		Total Count (All)	Strongly or somewhat disagree		Neither agree nor disagree		Somewhat or strongly agree		p-value
			N	%	N	%	N	%	
Years of GP experience	**Total**	107	9	8	12	11	86	80	
	GP Trainee	14	3	21	0	0	11	79	
	0 - 15 years post-CCT	44	3	7	7	16	34	77	
	15+ years post-CCT	49	3	6	5	10	41	82	0.07
GP Role	**Total**	107	9	8	12	11	86	79	
	Trainee GP	14	3	21	0	0	11	79	
	Qualified GP	93	6	6	12	13	75	79	0.04
Gender	**Total**	103	9	9	11	10	83	79	
	Male	42	4	9	6	14	32	74	
	Female	61	5	8	5	8	51	82	0.29
Location of practice	**Total**	107	9	8	12	11	86	79	
	London	36	2	5	3	8	31	82	
	Rest of UK	71	7	10	9	13	55	77	0.28
Ethnicity	**Total**	102	9	9	11	11	82	79	
	White	62	3	5	6	10	53	84	
	Non-white	40	6	15	5	12	29	71	0.09

2. For women who are at high risk of breast cancer, increase the frequency of screening

		Total Count (All)	Strongly or somewhat disagree		Neither agree nor disagree		Somewhat or strongly agree		p-value
			N	%	N	%	N	%	
Years of GP experience	**Total**	106	9	8	20	19	77	71	
	GP Trainee	13	2	14	1	7	10	71	
	0 - 15 years post-CCT	44	4	9	8	18	32	73	
	15+ years post-CCT	49	3	6	11	22	35	70	0.23
GP Role	**Total**	106	9	8	20	18	77	71	
	Trainee GP	13	2	14	1	7	10	71	
	Qualified GP	93	7	7	19	20	67	71	0.20
Gender	**Total**	102	9	9	18	17	75	71	
	Male	42	4	9	8	19	30	70	
	Female	60	5	8	10	16	45	73	0.46
Location of practice	**Total**	106	9	8	20	18	77	71	
	London	35	2	5	6	16	27	71	
	Rest of UK	71	7	10	14	20	50	70	0.35
Ethnicity	**Total**	101	9	9	19	18	73	70	
	White	62	3	5	13	21	46	73	
	Non-white	39	6	15	6	15	27	66	0.09

3. For women who are at high risk of breast cancer, increase the frequency of screening and start at an earlier age

		Total Count (All)	Strongly or somewhat disagree		Neither agree nor disagree		Somewhat or strongly agree		p-value
			N	%	N	%	N	%	
Years of GP experience	**Total**	107	8	7	19	18	80	74	
	GP Trainee	14	2	14	1	7	11	79	
	0 - 15 years post-CCT	44	3	7	7	16	34	77	
	15+ years post-CCT	49	3	6	11	22	35	70	0.20
GP Role	**Total**	107	8	7	19	17	80	73	
	Trainee GP	14	2	14	1	7	11	79	
	Qualified GP	93	6	6	18	19	69	73	0.18
Gender	**Total**	103	8	8	18	17	77	73	
	Male	42	5	12	8	19	29	67	
	Female	61	3	5	10	16	48	77	0.19
Location of practice	**Total**	107	8	7	19	17	80	73	
	London	36	2	5	6	16	28	74	
	Rest of UK	71	6	8	13	18	52	73	0.42
Ethnicity	**Total**	102	8	8	17	16	77	74	
	White	62	3	5	10	16	49	78	
	Non-white	40	5	12	7	17	28	68	0.17

4. For women who are at higher than average risk of breast cancer, increase the frequency of screening

			Strongly or somewhat disagree		Neither agree nor disagree		Somewhat or strongly agree		
		Total Count (All)	N	%	N	%	N	%	p-value
Years of GP experience	**Total**	104	6	6	32	30	66	61	
	GP Trainee	13	1	7	1	7	11	79	
	0 - 15 years post-CCT	43	3	7	13	30	27	61	
	15+ years post-CCT	48	2	4	18	36	28	56	0.11
GP Role	**Total**	104	6	6	32	29	66	61	
	Trainee GP	13	1	7	1	7	11	79	
	Qualified GP	91	5	5	31	33	55	58	0.08
Gender	**Total**	100	6	6	31	30	63	60	
	Male	42	2	5	16	37	24	56	
	Female	58	4	6	15	24	39	63	0.21
Location of practice	**Total**	104	6	6	32	29	66	61	
	London	35	2	5	11	29	22	58	
	Rest of UK	69	4	6	21	30	44	62	0.50
Ethnicity	**Total**	99	6	6	31	30	62	60	
	White	61	2	3	21	33	38	60	
	Non-white	38	4	10	10	24	24	59	0.14

5. **For women who are at higher than average risk of breast cancer, increase the frequency of screening and start at an earlier age**

		Total Count (All)	Strongly or somewhat disagree		Neither agree nor disagree		Somewhat or strongly agree		p-value
			N	%	N	%	N	%	
Years of GP experience	**Total**	104	7	6	31	29	66	61	
	GP Trainee	13	1	7	1	7	11	79	
	0 - 15 years post-CCT	43	3	7	13	30	27	61	
	15+ years post-CCT	48	3	6	17	34	28	56	0.14
GP Role	**Total**	104	7	6	31	28	66	61	
	Trainee GP	13	1	7	1	7	11	79	
	Qualified GP	91	6	6	30	32	55	58	0.09
Gender	**Total**	100	7	7	30	29	63	60	
	Male	42	4	9	13	30	25	58	
	Female	58	3	5	17	27	38	61	0.33
Location of practice	**Total**	104	7	6	31	28	66	61	
	London	36	3	8	11	29	22	58	
	Rest of UK	68	4	6	20	28	44	62	0.44
Ethnicity	**Total**	99	7	7	30	29	62	60	
	White	60	3	5	20	32	37	59	
	Non-white	39	4	10	10	24	25	61	0.25

6. For women who are at lower than average risk of breast cancer, start screening at a later age

		Total Count (All)	Strongly or somewhat disagree		Neither agree nor disagree		Somewhat or strongly agree		p-value
			N	%	N	%	N	%	
Years of GP experience	**Total**	104	43	40	41	38	20	19	
	GP Trainee	14	6	43	4	29	4	29	
	0 - 15 years post-CCT	43	20	45	17	39	6	14	
	15+ years post-CCT	47	17	34	20	40	10	20	0.22
GP Role	**Total**	104	43	39	41	38	20	18	
	Trainee GP	14	6	43	4	29	4	29	
	Qualified GP	90	37	39	37	39	16	17	0.27
Gender	**Total**	100	41	39	40	38	19	18	
	Male	42	15	35	18	42	9	21	
	Female	58	26	42	22	35	10	16	0.32
Location of practice	**Total**	104	43	39	41	38	20	18	
	London	36	15	39	17	45	4	11	
	Rest of UK	68	28	39	24	34	16	23	0.13
Ethnicity	**Total**	99	41	39	38	37	20	19	
	White	60	22	35	23	37	15	24	
	Non-white	39	19	46	15	37	5	12	0.14

7. For women who are at lower than average risk of breast cancer, decrease the frequency screening

		Total Count (All)	Strongly or somewhat disagree		Neither agree nor disagree		Somewhat or strongly agree		p-value
			N	%	N	%	N	%	
Years of GP experience	**Total**	104	35	32	46	43	23	21	
	GP Trainee	14	5	36	4	29	5	36	
	0 - 15 years post-CCT	43	16	36	21	48	6	14	
	15+ years post-CCT	47	14	28	21	42	12	24	0.13
GP Role	**Total**	104	35	32	46	42	23	21	
	Trainee GP	14	5	36	4	29	5	36	
	Qualified GP	90	30	32	42	44	18	19	0.16
Gender	**Total**	100	33	31	45	43	22	21	
	Male	42	9	21	23	53	10	23	
	Female	58	24	39	22	35	12	19	0.05
Location of practice	**Total**	104	35	32	46	42	23	21	
	London	36	14	37	18	47	4	11	
	Rest of UK	68	21	30	28	39	19	27	0.07
Ethnicity	**Total**	99	33	32	43	41	23	22	
	White	60	19	30	23	37	18	29	
	Non-white	39	14	34	20	49	5	12	0.07

8. For women who are at lower than average risk of breast cancer, decrease the frequency of screening and start at a later age

		Total Count (All)	Strongly or somewhat disagree		Neither agree nor disagree		Somewhat or strongly agree		p-value
			N	%	N	%	N	%	
Years of GP experience	**Total**	105	41	38	45	42	19	18	
	GP Trainee	14	5	36	4	29	5	36	
	0 - 15 years post-CCT	43	18	41	20	45	5	11	
	15+ years post-CCT	48	18	36	21	42	9	18	0.12
GP Role	**Total**	105	41	38	45	41	19	17	
	Trainee GP	14	5	36	4	29	5	36	
	Qualified GP	91	36	38	41	43	14	15	0.08
Gender	**Total**	101	39	37	44	42	18	17	
	Male	42	14	33	20	47	8	19	
	Female	59	25	40	24	39	10	16	0.33
Location of practice	**Total**	105	41	38	45	41	19	17	
	London	36	15	39	18	47	3	8	
	Rest of UK	69	26	37	27	38	16	23	0.08
Ethnicity	**Total**	100	39	38	42	40	19	18	
	White	61	23	37	23	37	15	24	
	Non-white	39	16	39	19	46	4	10	0.09

9. For women who are at much lower than average risk of breast cancer, do not offer breast screening

		Total Count (All)	Strongly or somewhat disagree		Neither agree nor disagree		Somewhat or strongly agree		p-value
			N	%	N	%	N	%	
Years of GP experience	**Total**	105	57	53	37	34	11	10	
	GP Trainee	14	7	50	3	21	4	29	
	0 - 15 years post-CCT	43	26	59	14	32	3	7	
	15+ years post-CCT	48	24	48	20	40	4	8	0.05
GP Role	**Total**	105	57	52	37	34	11	10	
	Trainee GP	14	7	50	3	21	4	29	
	Qualified GP	91	50	53	34	36	7	7	0.03
Gender	**Total**	101	56	53	34	32	11	10	
	Male	42	22	51	15	35	5	12	
	Female	59	34	55	19	31	6	10	0.43
Location of practice	**Total**	105	57	52	37	34	11	10	
	London	36	21	55	13	34	2	5	
	Rest of UK	69	36	51	24	34	9	13	0.24
Ethnicity	**Total**	100	55	53	34	33	11	11	
	White	61	31	49	21	33	9	14	
	Non-white	39	24	59	13	32	2	5	0.14

Table S3. Self-reported familiarity with PRS

			Not familiar (0-1)		Slightly to moderately familiar (2-4)		Very to extremely familiar (5-7)		
		Total Count (All)	N	%	N	%	N	%	p-value
Years of GP experience	**Total**	91	44	41	38	35	9	8	
	GP Trainee	9	5	36	3	21	1	7	
	0 - 15 years post-CCT	36	19	43	15	34	2	5	
	15+ years post-CCT	46	20	40	20	40	6	12	0.26
GP Role	**Total**	92	45	41	38	35	9	8	
	Trainee GP	9	5	36	3	21	1	7	
	Qualified GP	83	40	42	35	37	8	8	0.44
Gender	**Total**	88	43	41	36	34	9	9	
	Male	40	17	40	19	44	4	9	
	Female	48	26	42	17	27	5	8	0.25
Location of practice	**Total**	92	45	41	38	35	9	8	
	London	31	17	45	11	29	3	8	
	Rest of UK	61	28	39	27	38	6	8	0.35
Ethnicity	**Total**	87	41	39	37	36	9	9	
	White	50	23	37	22	35	5	8	
	Non-white	37	18	44	15	37	4	10	0.47

Table S4. Self-reported confidence communicating the difference between a polygenic and monogenic condition to a patient

		Total Count (All)	Not confident (0-1)		Slightly to moderately confident (2-4)		Very to extremely confident (5-7)		p-value
			N	%	N	%	N	%	
Years of GP experience	**Total**	101	22	20	57	53	22	20	
	GP Trainee	12	1	7	9	64	2	14	
	0 - 15 years post-CCT	41	6	14	27	61	8	18	
	15+ years post-CCT	48	15	30	21	42	12	24	0.04
GP Role	**Total**	102	23	21	57	52	22	20	
	Trainee GP	12	1	7	9	64	2	14	
	Qualified GP	90	22	23	48	51	20	21	0.16
Gender	**Total**	98	22	21	55	52	21	20	
	Male	41	10	23	22	51	9	21	
	Female	57	12	19	33	53	12	19	0.45
Location of practice	**Total**	102	23	21	57	52	22	20	
	London	35	7	18	22	58	6	16	
	Rest of UK	67	16	23	35	49	16	23	0.29
Ethnicity	**Total**	97	22	21	55	53	20	19	
	White	59	14	22	31	49	14	22	
	Non-white	38	8	20	24	59	6	15	0.27

Table S5: Self-reported confidence communicating the advantages and disadvantages of a personalised breast cancer risk assessment incorporating PRS to a patient

			Not confident (0-1)		Slightly to moderately confident (2-4)		Very to extremely confident (5-7)		
		Total Count (All)	N	%	N	%	N	%	p-value
Years of GP experience	**Total**	93	39	36	45	42	9	8	
	GP Trainee	11	4	29	6	43	1	7	
	0 - 15 years post-CCT	39	20	45	15	34	4	9	
	15+ years post-CCT	43	15	30	24	48	4	8	0.20
GP Role	**Total**	94	40	37	45	41	9	8	
	Trainee GP	11	4	29	6	43	1	7	
	Qualified GP	83	36	38	39	41	8	8	0.45
Gender	**Total**	90	37	35	45	43	8	8	
	Male	38	14	33	21	49	3	7	
	Female	52	23	37	24	39	5	8	0.35
Location of practice	**Total**	94	40	37	45	41	9	8	
	London	32	19	50	11	29	2	5	
	Rest of UK	62	21	30	34	48	7	10	0.03
Ethnicity	**Total**	89	37	36	43	41	9	9	
	White	52	21	33	26	41	5	8	
	Non-white	37	16	39	17	41	4	10	0.46

Table S6. Self-reported confidence communicating a personalised breast cancer risk assessment result as a 10 year absolute risk to a patient

		Total Count (All)	Not confident (0-1)		Slightly to moderately confident (2-4)		Very to extremely confident (5-7)		p-value
			N	%	N	%	N	%	
Years of GP experience	**Total**	105	33	31	50	46	22	20	
	GP Trainee	13	4	29	6	43	3	21	
	0 - 15 years post-CCT	44	14	32	21	48	9	20	
	15+ years post-CCT	48	15	30	23	46	10	20	0.33
GP Role	**Total**	105	33	30	50	46	22	20	
	Trainee GP	13	4	29	6	43	3	21	
	Qualified GP	92	29	31	44	46	19	20	0.49
Gender	**Total**	101	31	30	49	47	21	20	
	Male	41	14	33	18	42	9	21	
	Female	60	17	27	31	50	12	19	0.37
Location of practice	**Total**	105	33	30	50	46	22	20	
	London	37	14	37	17	45	6	16	
	Rest of UK	68	19	27	33	46	16	23	0.25
Ethnicity	**Total**	100	30	29	49	47	21	20	
	White	60	17	27	28	44	15	24	
	Non-white	40	13	32	21	51	6	15	0.24

Table S7. Perception of impact of personalised breast cancer risk assessment on patients

		Total Count (All)	Extremely negative (0-1)		Somewhat negative (2-3)		Somewhat positive (4-5)		Extremely positive (6-7)		p-value
			N	%	N	%	N	%	N	%	
Years of GP experience	**Total**	106	5	5	15	14	70	65	16	15	
	GP Trainee	13	1	7	1	7	9	64	2	14	
	0 - 15 years post-CCT	44	2	5	8	18	28	64	6	14	
	15+ years post-CCT	49	2	4	6	12	33	66	8	16	0.32
GP Role	**Total**	106	5	5	15	14	70	64	16	15	
	Trainee GP	13	1	7	1	7	9	64	2	14	
	Qualified GP	93	4	4	14	15	61	64	14	15	0.43
Gender	**Total**	102	4	4	14	13	68	65	16	15	
	Male	42	0	0	4	9	29	67	9	21	
	Female	60	4	6	10	16	39	63	7	11	0.08
Location of practice	**Total**	106	5	5	15	14	70	64	16	15	
	London	36	0	0	4	11	27	71	5	13	
	Rest of UK	70	5	7	11	15	43	61	11	15	0.15
Ethnicity	**Total**	101	4	4	14	13	67	64	16	15	
	White	62	3	5	11	17	42	67	6	10	
	Non-white	39	1	2	3	7	25	61	10	24	0.06

Table S8. Using the sliding scale below, please rate the impact you feel a personalised breast cancer risk assessment would have on your practice

		Total Count (All)	Extremely negative (0-1)		Somewhat negative (2-3)		Somewhat positive (4-5)		Extremely positive (6-7)		p-value
			N	%	N	%	N	%	N	%	
Years of GP experience	**Total**	105	6	6	34	31	59	55	6	6	
	GP Trainee	13	0	0	3	21	10	71	0	0	
	0 - 15 years post-CCT	44	3	7	19	43	20	45	2	5	
	15+ years post-CCT	48	3	6	12	24	29	58	4	8	0.10
GP Role	**Total**	105	6	6	34	31	59	54	6	6	
	Trainee GP	13	0	0	3	21	10	71	0	0	
	Qualified GP	92	6	6	31	33	49	52	6	6	0.18
Gender	**Total**	101	5	5	33	31	57	54	6	6	
	Male	42	1	2	15	35	24	56	2	5	
	Female	59	4	6	18	29	33	53	4	6	0.36
Location of practice	**Total**	105	6	6	34	31	59	54	6	6	
	London	36	0	0	12	32	20	53	4	11	
	Rest of UK	69	6	8	22	31	39	55	2	3	0.06
Ethnicity	**Total**	100	5	5	32	31	57	55	6	6	
	White	61	5	8	18	29	35	56	3	5	
	Non-white	39	0	0	14	34	22	54	3	7	0.14

Table S9. Categorisations used in questions about knowledge and attitudes

**Sliding scale groups for the four questions assessing knowledge**	• 0 – 1 'Not familiar' / 'Not confident'<br>• 2 – 4 'Slight to moderately familiar' / 'Slight to moderately confident'<br>• 5 – 7 'Very or extremely familiar'/ 'Very or extremely confident'
**Sliding scale groups for the two questions assessing impact**	• 0 – 1 'Extremely negative'<br>• 2 – 3 'Somewhat negative'<br>• 4 – 5 'Somewhat positive'<br>• 6 – 7 'Extremely positive'
**Likert-scale groups for the two questions about views on NHSBSP and future risk-stratified targeted screening approaches**	• 'Strongly agree or somewhat agree'<br>• 'Neither agree nor disagree'<br>• 'Strongly disagree or somewhat disagree'

Table S10. Categorisations used in questions about respondents' professional and sociodemographic characteristics

**Years of practice**	• 'GP Trainee'<br>• 0 – 15 years post-CCT<br>• '15+ years post-CCT'
**GP role**	• 'GP Trainee'<br>• 'GP Partner'<br>• 'Salaried', 'Locum' or 'Other GP'
**Location of practice**	• 'London'<br>• 'Rest of UK'
**Gender**	• 'Male'<br>• 'Female'
**Ethnicity**	• 'White'<br>• 'non-White'

***CCT = post-completion of GP Training**
